# Quantum ontology without speculation

**DOI:** 10.1007/s13194-020-00346-1

**Published:** 2021-01-30

**Authors:** Matthias Egg

**Affiliations:** Institute of Philosophy, University of Bern, Laenggassstrasse 49, CH-3012 Bern, Switzerland

**Keywords:** Effective realism, Ontology, Quantum mechanics, Spin, Underdetermination

## Abstract

Existing proposals concerning the ontology of quantum mechanics (QM) either involve speculation that goes beyond the scientific evidence or abandon realism about large parts of QM. This paper proposes a way out of this dilemma, by showing that QM as it is formulated in standard textbooks allows for a much more substantive ontological commitment than is usually acknowledged. For this purpose, I defend a non-fundamentalist approach to ontology, which is then applied to various aspects of QM. In particular, I will defend realism about spin, which has been viewed as a particularly hard case for the ontology of QM.

## Introduction

The relationship between quantum mechanics (QM) and ontology has always been a difficult one, and arguably, many of the difficulties stem from the so-called measurement problem (see Myrvold 2017 for a brief introduction and further references). The way in which the measurement problem has made quantum ontology difficult has, however, undergone a rather profound transformation during the history of QM. At least up to the 1960’s, when the cluster of views called “the Copenhagen interpretation” dominated the debate, the measurement problem manifested itself in the need to introduce some kind of split between the system described by QM and the classical world in which the results of measurements are observed. The part of the theory that was not concerned with the outcomes of measurements was then generally viewed in a non-realistic way, such that questions about the ontology of QM, in the sense of asking what the quantum formalism tells us about reality beyond measurements, were not given much consideration.

This situation changed with the advent of new ways of thinking about the measurement problem, associated with names like David Bohm, Hugh Everett III or John S. Bell. Being dissatisfied with the Copenhagen approach, these physicists sought to develop interpretations or versions of QM that did not depend on an arbitrary split between the quantum domain and the classical world, and could therefore be viewed as steps towards a coherent ontology of QM. It is customary to distinguish three classes of such proposals: First, theories supplementing the quantum formalism with additional variables (e.g., the de Broglie-Bohm theory), second, theories modifying the dynamics of QM so as to account for the disappearance of superpositions (e.g., spontaneous collapse models of the GRW type), and third, approaches modifying our understanding of the macroscopic world in such a way that it conforms to the unaltered formalism of QM (e.g., Everettian many-worlds theories). In this context, the measurement problem takes on a new role: The worry is no longer that questions about quantum ontology are somehow illegitimate, but since the proposals put forward in response to these questions offer radically different (but empirically equivalent) pictures of reality, it becomes hard to justify a commitment to any particular one of them. This is still the situation with which we are confronted today: although the different proposals have progressively matured in the last few decades, we do not seem to approach any consensus about which one of them is to be preferred as a guide to the ontology of QM.

Another way to describe this predicament is to say that the choice between different quantum ontologies is *underdetermined* by the scientific evidence (Cordero^2001^; Lyre 2010; Lewis 2016, ch. 3). More precisely, it is helpful to stress that there are actually two layers of underdetermination involved. The first layer is the underdetermination just described, obtaining between the different responses to the measurement problem. These are sometimes called different “interpretations of QM”, but it is more appropriate to view them as predictively equivalent rival theories, which makes this an instance of the well-known problem of *underdetermination of theory by evidence* (Acuña 2019). The second layer of underdetermination is less well known, but it clearly appears in those recent debates on quantum ontology that are concerned with the question whether (and if so, how) quantum theories should be supplemented by a so-called *primitive ontology* (PO), that is, postulates about matter in ordinary space or space-time over and above what is contained in the standard formalism of QM (see Allori 2015 for an overview). What these controversies show is that each of the theories constituting the first layer of underdetermination is in turn compatible with multiple ontological options. The most famous case is the GRW theory, of which there is a version without PO (usually called “GRW0”), a version supplemented with a matter density in space (“GRWm”) and a version supplemented with flash-like events in space-time (“GRWf”).^1^ Taken together, the two layers of underdetermination generate a considerable number of ontological options compatible with the empirical basis of QM.

The problem of underdetermination underlies the notion of *speculation* appearing in the title of this paper. Accordingly, ontological content of a theory counts as speculative if it is subject to such underdetermination between scientifically serious alternatives. The restriction to *scientifically serious* alternatives is important, because otherwise, *any* ontological claim could be classified as speculative. As Stanford (2001, S2–S4) argues, underdetermination should not be taken seriously if it merely collapses the issue into a general philosophical problem, such as responding to Cartesian skepticism or to a radical agnosticism about all theoretical claims. Underdetermination only has argumentative bite if it presents us with a distinct challenge specific to the domain under discussion.^2^ Given what I just said about the origin of the various proposals for quantum ontology, this seems to be the case here.

Against this backdrop, the main aim of the present paper is to show that, despite the presence of serious underdetermination, a substantive non-speculative ontology of QM is possible. The difficulty of this task is brought to light in the review of hitherto existing proposals given in Section 2. As I will argue, such proposals are either too speculative or not sufficiently ontological. The key to overcoming this dilemma is the acknowledgment that QM is a non-fundamental theory and that consequently, quantum ontology is inherently non-fundamental as well. However, given the time-honored association of ontology with fundamentality, the very idea of non-fundamental ontology requires some defense, which will be provided in Section 3. Once this is achieved, the idea can be applied to concrete aspects of QM. Section 4 thus argues for a non-speculative ontological commitment to the quantum property of spin, a case which has been claimed to be particularly troubling for any realistic approach to QM. Further examples of ontological commitment will be discussed in Section 5, which also responds to a number of objections that have been raised against this kind of quantum ontology.

## The unsuccessful quest for a non-speculative quantum ontology

The situation sketched in the introduction generates a dilemma for anyone seeking to draw ontological information out of QM: either opt for one particular version of QM, but then you face the above-described problem of underdetermination; or limit your ontological commitment to some core content of QM unaffected by the underdetermination between its different versions, but this seems to lead you back to the somewhat anti-ontological “Copenhagen” way of thinking about QM.

Various ways of dealing with this dilemma have been proposed in the literature. Among those who embrace its first horn, two different ways of addressing the problem need to be distinguished. First, one can be concerned with the ontology of QM without worrying about underdetermination, because one views the task of ontology as spelling out *how the world could be*, not as issuing claims about *how the world is*. A clear exemplification of that spirit is the exploration of various PO models (some of which quite implausible, as admitted by the authors themselves) in Allori et al. (2014). I grant that there is value in this kind of work, but it is not the understanding of “ontology” with which I am concerned in this paper, because it is an ontology that celebrates (rather than avoids) speculation.

The second way of embracing the first horn takes itself to be non-speculative, because it includes the conviction that ultimately, there is no underdetermination. Although adherents of this approach usually admit that there is some empirical equivalence between the different versions of QM, they hold that one particular version is so much better than its alternatives that opting for it does not amount to speculation at all, but is simply what any scientifically reasonable person should do. It will suffice to mention two of the most outspoken representatives of this view.^3^ On the one hand, Wallace (2012) famously advocates the Everett interpretation as *the* straightforward way to approach QM. In a recent paper (Wallace 2020), he explicitly criticizes the claim that there is an underdetermination here, because the Everett approach is the only one that takes the whole framework of QM seriously, whereas its competitors (in particular, Bohmian mechanics and the GRW theory) are almost exclusively concerned with a small subdomain of QM (namely, non-relativistic particle mechanics). On the other hand, Bricmont (2016) argues that none of the alternative versions of QM (Everett included) matches the Bohmian approach in terms of clarity and explanatory power, which is therefore the only way to really *understand* QM. Ironically, then, by their very efforts to demonstrate the absence of underdetermination, Wallace and Bricmont clearly show that one can with good reasons hold on to one of at least two fundamentally incompatible versions of QM, which is to say that any such choice is speculative in the sense employed here.

When we now move towards the second horn of the dilemma, we encounter the problem of the multifarious uses of the term “realism”. Several authors have argued that one can keep away from the first horn, while still being a “realist” about QM. The confusing thing about this is that some of these proposals have commonly been viewed (quite naturally, to my mind) as *alternatives* to realism. So Healey (2020) now advocates a “pragmatist quantum realism”, and even a QBist like Fuchs (2017) insists on being a kind of realist.^4^ Whatever the merits of such unorthodox types of realism might be, they are quite clearly irrelevant to our present discussion, insofar as they share what can be called a *non-representationalist* approach to QM. This means two things: First, these authors take the quantum formalism to serve some other purpose than to represent elements of reality (e.g., to provide objective guidance for our beliefs). Additionally (and in contrast to some representationalists who share this attitude towards the *standard* quantum formalism), they refuse to propose any alternative formalism that *does* represent elements of reality, so as to account for the success of QM.^5^ In other words, they reject the very idea of quantum ontology and thereby fall prey to the second horn of the dilemma.

The same applies (although less obviously) to the information-theoretic approach to QM advocated by Bub (2016). I say less obviously, because this approach can be given a realist (even representationalist) interpretation, along the lines of *information-theoretic structural realism*, as developed by Ladyman and Ross (2007, 2013). In conjunction with their attempt to dissolve the measurement problem (Ladyman and Ross 2007, subsec. 3.7.4), one might hope that they succeed in navigating between the two horns of the dilemma. However, I have recently shown that such hopes are unfounded, because Ladyman and Ross’s allegiance to Niels Bohr’s views on QM pushes them too far towards the second horn (Egg 2019).

This leaves us with responses to the dilemma that rely on some kind of *selective realism*. These are set within a representationalist understanding of QM, but seek to delimit the resulting ontological commitments so as to avoid speculation. The question then becomes whether the resulting commitments are substantive enough to constitute an interesting quantum ontology. The answer is clearly negative in the case of Hoefer (2020), who essentially excludes the entire class of distinctively quantum theoretical claims from realist (and, consequently, representationalist) treatment. By contrast, Cordero (2001) and Saatsi (2019, 2020) recognize the impressive empirical success of QM as a strong motivation for some kind of representationalism in the quantum domain, but the ontological payoff of their approaches remains unclear. As we will see in Section 4, Saatsi dissociates the representational role of quantum notions from what he calls *truth-content realism* by denying that we are able to identify the referents of these notions in a non-speculative way. Accordingly, his *progress realism* remains silent on matters of quantum ontology. Cordero is more optimistic and even gives some concrete examples of what might constitute a non-speculative ontology of QM, but Callender (2020) has recently accused these examples of either not being genuinely quantum physical or of being subject to underdetermination (and therefore being speculative) after all. It thus seems that we are still missing a convincing proposal for a non-speculative quantum ontology.

## Non-fundamental ontology

As will become clear in Section 5, I actually think that much of Callender’s criticism of Cordero’s proposal is unjustified. In that sense, my own proposal is very close to Cordero’s, and I am happy to adopt the label Callender uses for designating its central element: “textbook quantum mechanics” (henceforth TQM). The key idea is that ontology should be informed by our best current theories^6^ and that what makes QM one of our best (i.e. empirically most successful) theories is not any of its ontologically kosher (speculative) formulations, but the somewhat messy and recipe-like form in which it appears in textbooks. Hence TQM should be taken ontologically seriously.

Given the wide variety of QM textbooks on the market, TQM is obviously not a precisely defined set of descriptions or models, let alone a clearly circumscribed theory. Nevertheless, there are several uncontroversial examples of scientific achievements that any version of QM must be able to reproduce. A considerable part of my case for a non-speculative quantum ontology, understood as an ontology of TQM, will therefore consist in a discussion of such examples (see Sections 4 and 5). But the first task is to introduce the most important general features of my approach and to dispel some initial worries that it might generate.

### Effective realism about textbook quantum mechanics

To many readers, TQM will seem like the last place to look for a serious ontology. After all (they will say), the ontological confusion prevalent in QM textbooks was one of the main reasons for developing the different alternative approaches to QM, so turning back to TQM is the very opposite of ontological progress. This way of thinking, however, rests on a failure to properly distinguish ontology from *fundamental* ontology. I fully agree that TQM does not offer us much guidance with respect to the latter (mainly because it does not solve the measurement problem), but this should not bother anyone who is aware that QM, understood as the kind of non-relativistic particle theory with which most of the debate referenced in Section 2 is concerned, is not a fundamental theory anyway. Although QM is sometimes loosely classified as part of “fundamental physics”, this can only mean that QM is fundamental *relative to* other parts of physics, for example condensed matter physics. However, the complaint that TQM is no guide to ontology stems from a preoccupation with fundamental ontology in an *absolute* sense, viz. an account of the basic building blocks of reality.^7^ The kind of quantum ontology I am going to develop here is not fundamental in this sense.

This ties in with the current trend in the philosophy of quantum field theory (QFT) to question what Williams (2019, 233) calls “the standard interpreters’ quixotic focus on fundamental structure”. As far as relative fundamentality is concerned, everyone agrees that QFT is more fundamental than (non-relativistic) QM, but there is a growing awareness that it contradicts the very nature of QFT to regard it as fundamental in an absolute sense. Instead, it should be regarded as an *effective* theory, that is, as a low-energy limit of a more fundamental theory, just as QM is the low-energy limit of QFT.^8^ Building on earlier work by David Wallace, Williams (2019) convincingly argues that QFT’s non-fundamental character should not stop us from drawing ontological lessons from it. In the same spirit, I maintain that we should treat QM as a source of information about (non-fundamental or effective) ontology, and not saddle it with any pretence of fundamentality.^9^

But what is the content of such an ontology? In the absence of a proper solution to the measurement problem, does TQM provide us with anything over and above a mathematical formalism and some rules on how to extract empirical predictions from it? To see how it does, one needs to realize that questions about the ontology of effective theories must be answered in *functional* terms. They cannot be answered by any reference to the *nature* of their theoretical posits, insofar as that would require knowledge about how these posits emerge from a fundamental theory, which is just what the effective theory does not provide. Instead, the posits of an effective theory are characterized by what they *do* (effectively), rather than by what they *are* (fundamentally). One might object that such a shift from *being* to *doing* takes us out of the domain of ontology. This objection leads us into a complex of meta-ontological disagreements, the proper treatment of which is beyond the scope of this paper. Let me just state that the approach to ontology taken in this paper is a functionalist one, which differs from the more traditional fundamentalist approach by acknowledging the value of spelling out what *X* does in response to the question of what *X* is. It seems to me that from a scientific perspective, this approach is far better motivated than its alternative, because it takes seriously the potential, but also the limitations of current, non-speculative science in providing us with an answer to the question of what there is. Our best scientific theories happen to be effective theories and these come with a functional ontology.

The notion of a functional ontology and the way in which such an ontology is present in TQM will become clearer (and hopefully more attractive) when we look at concrete examples. I will therefore return to these meta-ontological considerations in Section 5.3, where I will draw what I take to be the main lesson from the examples discussed in the present paper, regarding the question of how to think about ontology. In the meantime, the following general observation about TQM might serve as a first illustration: Many readers of QM textbooks will be familiar with the frustration of hearing much about “quantum systems” without ever being told *what a quantum system is*. The frustration is usually appeased by realizing that this does not really matter, because “quantum system” is merely a placeholder for *whatever it is that has a certain quantum state*. From a fundamentalist point of view, of course, the notion of a quantum state is no less obscure than “quantum system”, but as far as functional ontology is concerned, the descriptions given by TQM are very detailed and precise: State vectors (or wave functions) codify the behaviour that quantum systems display in virtue of their quantum states in given experimental situations. This is the sense in which the ontology of quantum states is given by what they *do*, namely to bring about specific kinds of behaviour in the quantum systems that are in those states.

### A *reductio* for effective realism?

Another worry about attributing substantive ontological content to effective theories is that it might lead to an unacceptable proliferation of ontological commitments. To use an infamous example, how can the effective ontologist avoid admitting that there is phlogiston, given that phlogiston theory effectively predicted and explained various phenomena? The answer is that it takes more than some empirical successes (impressive though they may be) to make for an effective theory. What marks a theory as *effective* is that it successfully deals with all the phenomena within a natural domain, where “natural” is usually spelled out in terms of a physical parameter (energy, for example) which needs to be in a certain range for the theory to be applicable. Phlogiston theory was abandoned because it did not meet that requirement.^10^

A more problematic example for the effective ontologist is the case of Newtonian gravity. Indeed, Ruetsche (2018) uses this very example in a kind of *reductio* argument against Williams’s effective realism (the view that we should be committed to the ontology of our effective theories). To put it briefly (and much less elegantly than Ruetsche): effective realism entails ontological commitment to gravitational forces, but general relativity tells us that gravity is not a force, hence effective realism is problematic. My response to this case is admittedly controversial and a full defense of it is beyond the scope of this paper, but the following remarks should at least make room for the idea that the reality of gravitational forces is not as indefensible as Ruetsche supposes.

My first observation in favor of this idea is just the old no-miracles argument for scientific realism: the impressive empirical success of Newton’s theory of gravity *prima facie* justifies the belief that its central posits (namely gravitational forces) exist. And in contrast to the phlogiston case (and other examples standardly employed by antirealists to counter the no-miracles argument), the success of Newtonian gravity is not confined to some limited historical period or to the simplistic applications found in introductory textbooks. As Bokulich (2016) shows, Newtonian gravity is indispensable to significant parts of cutting-edge contemporary science, for example in physical oceanography.^11^ Now Bokulich herself does not endorse realism about gravitational forces and criticises Wilson (2007) for doing so, because “the explanatory power of a posit does not automatically license a realist conclusion” (Bokulich 2016, 275). Although I’m on Wilson’s side in this controversy, I admit that there might be reasons to reject the inference from explanatory power to realism despite its *prima facie* plausibility. I will now examine those reasons, insofar as they are relevant to Ruetsche’s criticism.

Traditionally, resistance to realism has often been specifically concerned with unobservable entities. However, we do not need to dwell on the question whether gravitational forces are observable (see Wilson 2007, sec. 3 for an affirmative answer). For if gravitational forces are unobservable in any problematic sense, then so is spacetime curvature. And since Ruetsche’s argument is premised on the assumption that, according to general relativity, gravitational effects are actually a consequence of spacetime curvature (rather than produced by gravitational forces), lack of observability cannot be what underlies Ruetsche’s non-realism about such forces.

Another traditional source of suspicion regarding gravitational forces concerns their supposed action at a distance. Newton himself famously spoke of a “great absurdity” when commenting on the idea “that one body may act upon another at a distance through a vacuum, without the mediation of anything else, by and through which their action and force may be conveyed from one to another”.^12^ It is not clear, however, that the rejection of action at a distance should undermine realism about forces. Even if one adopts a field-theoretic perspective on Newtonian gravity, which does away with action at a distance, one can regard gravitational forces as (perfectly real) effects of the field, in just the same way as we standardly think of electromagnetic forces in classical electrodynamics.^13^

Presumably, Ruetsche’s reason to resist realism about gravitational forces is that they are absent from our most fundamental theory of gravity (i.e., general relativity). Yet this alone cannot be sufficient, unless one were prepared to abandon realism about all of science except fundamental physics. Since this would be a very unattractive consequence, let us take a closer look at the move from a diagnosis of non-fundamentality to a denial of reality. For concreteness, consider viruses as examples of objects whose reality is well established, although they do not appear in fundamental physics. Before looking at the issue of non-fundamentality in more detail, let me set aside another issue that might motivate a difference in attitude concerning viruses compared to gravitational forces. It might seem that a kind of no-miracles argument for realism is much better justified in the case of viruses, because we possess much more knowledge of their properties than we do in the case of gravitational forces. This has to do with the fact that viruses are much more complex entities than forces and that they are responsible for a much larger variety of phenomena than gravitational forces. In response, I would first like to point out that the empirical success which fuels the no-miracles argument is not only a matter of variety of explained phenomena. Other dimensions of success matter as well, for example, reliability and accuracy of predictions. And in that respect, scientific applications involving gravitational forces are often more successful than the ones involving viruses (compare our ability to predict tidal phenomena to our ability to predict the development of diseases). Second, even if one grants that our knowledge of viruses yields more empirical success than our knowledge of gravitational forces, then a similar argument as the one concerning (un-)observability applies: To the extent that one deems the scientific payoff of our knowledge about gravitational forces too limited to justify realism, one should also abstain from realism about spacetime curvature, because it is similarly limited. Indeed, Bokulich (2016, 273) convincingly argues that invoking the heavy machinery of general relativity instead of Newtonian gravity would, in many cases, result in inferior explanations.

Be that as it may, the usual rationale for ascribing reality to viruses while withholding it from gravitational forces is that we imagine viruses to be composed of entities that do appear in fundamental physics (namely electrons and quarks), while gravitational forces are not composed of anything that is mentioned in general relativity. Instead, general relativity explains gravitational effects by something (namely spacetime curvature) that is not even in the same category as a force. However, it should be well known that the supposed composition relation between elementary particles and larger objects (such as viruses) is highly non-trivial and that the “particles” of fundamental physics in fact have so little in common with ordinary objects that it becomes doubtful to even place them in the same category (see, e.g. Healey 2013). There are, of course, theoretical accounts of how certain configurations of quarks and electrons can give rise to viruses, but the same is true for the relationship between spacetime curvature and gravitational forces. The latter relationship is even straightforward enough to be included in an introductory course on general relativity, while the theoretical relationship between quarks, electrons and viruses depends on a rather complex combination of elements from quantum chromodynamics, nuclear physics, atomic physics and quantum chemistry. I therefore struggle to understand how one can claim that general relativity somehow excludes the reality of gravitational forces without admitting that the reality of viruses is likewise “excluded” by the standard model of particle physics.

As a last attempt to drive a wedge between the ontological status of viruses and gravitational forces, respectively, one might claim that the composition of elementary particles, although highly non-trivial and described by a patchwork of various theories, gives rise to actual viruses, whereas the Newtonian limit of general relativity, although comparatively simple mathematically, only gives rise to a certain structure that has the *appearance* of a gravitational force, but *is not actually* such a force. To respond to this point, one needs to remember the meta-ontological lesson of effective realism introduced in the previous subsection. According to the functionalist understanding of ontology that I am defending throughout the present paper, what it is to *be a force* is nothing over and above functioning *like a force* (in all relevant respects). In other words, to claim that what appears (to a very good approximation) in the Newtonian limit of general relativity only *appears* like a force without *being* a force is to insist on a difference that does not make a difference, which can only be justified by begging the question against effective realism.

To repeat, I do not expect the foregoing remarks to convince everyone of the reality of gravitational forces, but at least they should suffice to shift the burden of proof onto those who think that realism about gravitational forces is absurd, while realism about viruses is legitimate.

## How to be a realist about spin

### The problem with spin

To get a better grasp of the content and the justification of the quantum ontology I propose, it will be useful to consider an example that has been argued to pose particularly hard problems for any realism that seeks to find ontological content in TQM. As Saatsi (2020) demonstrates, the quantum notion of spin, while being crucially involved in many of the most impressive explanatory and predictive successes of QM, is plagued by a serious underdetermination in terms of what the notion refers to according to the various versions of QM. I will now argue that this kind of underdetermination is not one that should deter us from realism about spin.

First, I should clarify what is (and is not) meant by “realism about spin”. There is a sense in which such a realism is relatively uncontroversial: if “spin” is understood as just the state-independent property according to which, for example, “electrons have spin 1/2 and W bosons have spin 1”, then spin is on a par with other state-independent properties like mass and charge, whose reality is rarely disputed. Such a modest realism about spin would hardly call for a philosophical defense. On the other hand, “realism about spin” could also designate a thoroughly indefensible position, namely the view that the spin *component* along any given axis possesses a definite value at all times. This would contradict the uncertainty relations between spin components, which are part of any version of QM. To be viable, realism about spin components must incorporate these relations, and this leads to my positive proposal of what should be meant by “realism about spin”: it is a realism about spin states (and their components) *insofar as they are assigned to quantum systems by TQM*. In other words, the claim is that the spin part of the quantum mechanical wave function refers to a real physical property.^14^ It is easy to see how this understanding of realism generates the problem of underdetermination to which Saatsi draws our attention: since the ontological status of the wave function is highly contested among the different versions of QM, the status of spin is likewise contested, hence realism with respect to it seems unable to avoid speculation.

To be sure, Saatsi himself thinks that a kind of realism about spin is well motivated, indeed almost unavoidable:

In relation to key realist criteria, spin surely ticks all the boxes, by virtue of being deeply explanatory, unifying, and even effectively manipulable. Hence, we should be realists about spin, as much as we are realists about any theoretical notion. (Saatsi 2020, 41)

Nevertheless, Saatsi takes the example of spin to undermine central knowledge claims about quantum ontology (encapsulated in what he calls *truth-content realism*), because in his view, such claims cannot stay silent about the speculative (in his terminology: “deeply” metaphysical) aspects of QM:

The challenge to truth-content realism is that it seems forced to buy into “deeply” metaphysical assumptions—assumptions that are epistemologically unwarranted by the realist lights—in trying to spell out what we claim to know about, e.g., silver atoms in a Stern-Gerlach machine. (47–48)

In line with the argument of the previous section, my initial response to this challenge is the simple reminder that surely TQM gives us *some* knowledge about what happens in a Stern-Gerlach machine without buying into any deep metaphysics. In fact, it is precisely this kind of functional knowledge that yields the explanatory and manipulative successes which, as we just saw, motivate even Saatsi to adopt some kind of realism about spin. Now one might argue that this kind of knowledge is too shallow for truth-content realism, because it only concerns aspects of QM (namely, its mathematical formalism and its empirical predictions) with which few antirealists would disagree. However, this would be to neglect the various ways (nicely summarised in Saatsi 2020, sec. 3.2) in which spin accounts for a wide range of phenomena in physics and other sciences, by means of descriptions that widely exceed the realm of our observations and yet do not depend on specifying *what spin is* in terms of fundamental ontology, but only in terms of *what it does*. For example, the impressive applications of nuclear magnetic resonance (NMR) surely depend on actual and precise (but non-fundamental) *knowledge* about nuclear spin and its behaviour in response to external magnetic fields. An important part of this knowledge comes from interpreting the formalism of TQM as a (functional, if not even causal) description of how physical systems in virtue of their spin states interact with their surroundings (and not from any “deeply” metaphysical description of spin). Of course, one can always insist on an antirealist construal of such knowledge claims, but then there is nothing special about spin, and the argument would boil down to a rather uninteresting *a priori* dismissal of truth-content realism.

At this point, the disagreement between Saatsi and myself may begin to look unsubstantial, for it seems to rest on the mere fact that my notion of “knowledge” and “truth-content” is somewhat broader than his. In other words, it seems that we both appreciate the value of an effective realism about TQM as introduced in Section 3, but merely differ in whether it should be classified as an instance of truth-content realism or rather some weaker kind of realism (such as Saatsi’s *progress realism*).

However, there is more to Saatsi’s criticism than just the suspicion that the knowledge given by TQM is too shallow to satisfy the demands of truth-content realism. His main point is that even in its shallowness, the content of TQM is subverted by the underdetermination that kicks in as soon as one tries to give a realist account of that content:

Spelling out the workings of a Stern-Gerlach machine along these lines leads to specific realist accounts of what it *means* to attribute spin-1/2 to an electron or a silver atom, so as to explain the Stern-Gerlach experiment. These accounts diverge radically from a face-value textbook reading of quantum mechanics, according to which electrons have an *intrinsic (non-classical) property* of spin-1/2, which silver atoms also have due to the way in which electron spins quantum physically combine to yield the atom’s total spin, which affords the atom the property of intrinsic magnetic moment that interacts with the magnetic field to yield the observed outcome (after a measurement “collapse”). (47)

If I now attempt to show that, despite this apparent divergence between TQM and some realistic accounts of spin, there is a core content of realism about spin on which all such accounts agree, I cannot discuss all of them explicitly. To keep things manageable, I will focus the discussion on those proposals which differ most starkly in their ontological commitments. It can then be reasonably expected that a similar treatment will be able to accommodate less extreme proposals as well.

### Against underdetermination about the ontology of spin

Let us start with the question what it is that *has* spin. TQM typically describes spin as a property of *particles*, but then adds that “particles” should not be understood in the classical sense of little bits of matter that always follow definite trajectories. Indeed, unless and until a measurement is performed, particles in TQM typically do not have definite positions, and their state is completely described by the wave function, which also incorporates the description of their spin state. Turning from TQM to the ontologically precise versions of QM, this pre-measurement picture is retained in some variants (those that subscribe to some form of quantum state monism), but is sharply at odds with others, in particular those which postulate a primitive ontology (PO). As an example, consider the minimalist version of Bohmian mechanics, according to which the only properties that particles possess are their positions.^15^ On this view, the wave function (including its spin part) is usually attributed a nomological (rather than ontological) role (Goldstein and Zanghì 2013). Add to this a Humean outlook, according to which laws are nothing more than convenient summaries of regularities that obtain in the mosaic of particular facts, and you arrive at the kind of “Super-Humeanism” defended by Esfeld and Deckert (Esfeld and Deckert 2018b), where spin no longer seems to be a real property. Nor is this a peculiarity of Bohmian mechanics; the same move is possible (though somewhat less motivated, see Egg and Esfeld 2015) in PO versions of the GRW approach. It thus seems as if the reality of spin could not be part of a non-speculative realism about QM, due to an underdetermination between versions of the theory that include it and versions that do not (Vickers 2020, sec. 5).

But as it turns out, this impression is false. To see why, let us first recall the basic motivation for realism about spin, namely the notion’s explanatory and predictive power, and let us note that this is not disputed by any serious account of QM. More specifically, I do not know of any minimalist Bohmian explanation of spin-related phenomena that seeks to *replace* what TQM teaches about spin. Rather, such explanations seek to *recover* the TQM account and to provide a firm ontological basis for it (see, e.g., Norsen 2014). To better understand what this means, it is instructive to look at a recent exchange between Wilson (2018) and Esfeld and Deckert (2018a), in which Wilson accuses Super-Humeanism of being a kind of instrumentalism about “theoretical entities such as wavefunctions, fields, and spin properties” (429). Due to the undeniable explanatory role of such entities, this is problematic for anyone who accepts the no-miracles intuition (or, as Wilson (430) phrases it, the intuition “that explanation must be factive”). As good scientific realists, Esfeld and Deckert naturally share this intuition and defend themselves against the charge of instrumentalism by insisting that their view does not *eliminate* these features of the world, but *locates* them “in configurations of matter points and their change” (447). Since this is done by identifying their functional role, to say that these features are real (as opposed to mere instruments for prediction) is essentially to adopt the kind of effective realism I sketched in Section 3.

Still, the idea of *locating* spin in configurations of matter points and their change suggests that one might be a kind of realist about spin and yet insist that one’s ontology consists of nothing but these fundamental matter points, and does not include spin. For reasons discussed in Section 3, I find this use of the term “ontology” quite unfortunate, as it renders dubious the very idea of an ontology of QM (or of any other non-fundamental theory). But even if one accepts, for the sake of the argument, the proposal to equate ontology with fundamental ontology, it still does not follow that the supposed absence of spin from the ontology of Super-Humeanism creates an interesting underdetermination between different versions of QM. For according to this proposal, spin is denied ontological status on the sole ground that it is not fundamental, hence the alleged underdetermination affects *all* non-fundamental entities (from viruses to baseballs and beyond), which are likewise *located* in configurations of matter points and their change. Following Stanford’s advice cited in Section 1, such general underdetermination should not be taken seriously in philosophy of science.

As a last attempt to find some relevant divergence between what different versions of QM tell us about the ontology of spin, one may argue that non-fundamentality is not the only reason to exclude spin from one’s ontology. Since spin is part of the quantum state and since the latter has a nomological (or quasi-nomological), but not an ontological status in some PO versions of QM (including Super-Humeanism), there might be a specifically quantum mechanical type of underdetermination with regard to spin after all. I will respond to this worry in the context of what I call the *nothing-there* objection in Section 5.2.

### Against underdetermination about wave function collapse

The second locus of radical divergence between different versions of QM concerns the persistence of superpositions. According to so-called “collapse-theories”, there is a physical process in the evolution of the quantum state that eliminates superpositions and thereby accounts for the uniqueness of measurement outcomes, whereas “no-collapse-theories” describe superpositions of quantum states as persisting and tell some other story about why we do not observe them directly. For a clear example of this contrast, let us look at the GRW (collapse) theory and the Everettian (many-worlds) interpretation as the main representatives of these two camps, and let us consider how they differ in their account of a Stern-Gerlach experiment that measures the spin of a particle whose spin state is in a superposition with respect to the direction along which the apparatus is oriented.

The GRW theory can be viewed as an improvement on TQM with its ad-hoc notion of “collapse upon measurement”, by providing a clear account of when a “measurement” takes place and causes the quantum superposition to disappear. In the Stern-Gerlach case, this happens (with probability close to 1) when the particle, having passed the inhomogeneous magnetic field, interacts with the device that is supposed to measure whether it was deflected upward or downward by the magnet. (Strictly speaking, the device does not measure this, because there is no “upward *or* downward” prior to wave-function collapse. Up to this point, GRW and Everett tell exactly the same story about what happens in the experiment.) The collapse then ensures that there is no superposition of measurement outcomes, thereby accounting for our experience that measurements have definite results.^16^

According to the many-worlds theory, the superposition of states persists until and beyond the end of the experiment. Contrary to the collapse story of GRW (and TQM), our experience of a unique measurement result (e.g., “spin up”) is not due to the non-occurrence of the alternative result (“spin down”), but due to the fact that this alternative result obtains in a parallel branch of the universe that is (due to decoherence, for all practical purposes) observationally inaccessible from our branch.

So what kind of underdetermination does this difference between a collapse and a no-collapse view generate? On a certain reading of the many-worlds theory, it would seem that our knowledge about measurement outcomes is quite radically underdetermined: we should not believe a straightforward proposition like “the result is ‘spin up’ and *not* ‘spin down’ ”, because on that reading of Everett, the correct thing to say would be “the result is ‘spin up’ and *also* ‘spin down’ ”. However, this way of putting things can quite easily be dismissed. First, given the ubiquity of quantum processes in nature, this kind of underdetermination is again of no specific interest for philosophy of science: it also affects everyday claims like “my eyes are blue and not brown”, for if Everett is right, then surely there exists some brown-eyed copy of myself in another branch of the universe. And anyway, there is a much more plausible way of understanding such statements in an Everettian context, by indexing them to the branch in which they are uttered (see Wallace 2012, ch. 7 for details). In that way, the Everettian and the collapse theorist will agree not only on a statement like “the result is ‘spin up’ ”, but also on the statement “the result is *not* ‘spin down’ ” (within the branch they are in).

This reduces the scope of the underdetermination problem to a rather limited part of the Stern-Gerlach process. We have seen above that the two accounts agree in their description of what happens before the collapse. We now realize that there is a plausible sense in which they also agree about what obtains once decoherence has done its job of generating (according to the many-worlds story) different branches with a unique measurement result in each of them.^17^ And with respect to what happens in between these two stages, the remaining underdetermination does not look all that worrying, because it is in principle (and—to an increasing degree—in practice) open to empirical investigation (Bassi et al. 2013). It is therefore not overly optimistic to claim that future research will provide us with quite specific knowledge about the process in which a unique measurement outcome emerges from a superposed state in a Stern-Gerlach experiment.

Two caveats about this optimism are in order. First, there will be some inevitable vagueness in the knowledge just mentioned, regardless of whether the empirical inquiry turns out in favour of collapse or of no-collapse theories. In the former case, the vagueness will be due to the inherently stochastic character of the GRW dynamics, in the latter, it will stem from the non-fundamental nature of the branching process, which entails a certain context-dependence in the identification of decohering branches. But this is just what we should expect within the framework of a non-fundamental ontology, as discussed in Section 3. The second caveat concerns completeness claims that are sometimes made in connection with scientific ontology, to the effect that there are no parts of reality outside of what is described by science. The serious possibility of there being many worlds implies that the above response to the underdetermination problem can only succeed in a branch-relative sense, hence there is no non-speculative justification for any claim that the knowledge provided by TQM exhausts even the non-relativistic domain. In other words, while we can be sure that the results of our spin measurements are real, we cannot assert that seemingly incompatible alternative results are not also real (in a non-branch-relative sense).

## Discussion of further examples and objections

### Callender’s objections

By looking at some other parts of TQM, this section seeks to give further substance to the idea of a non-speculative quantum ontology and to dispel some objections raised by Callender (2020, sec. 4.5) against Cordero’s (2001) version of realism about TQM. After setting aside examples that, although mentioned in some QM textbooks, are not truly quantum mechanical (such as basic claims about the shape and structure of the water molecule), Callender discusses three classes of phenomena to which a realist about TQM may want to commit himself ontologically:

1. semi-classical orbits (e.g., in the hydrogen atom),
2. quantum tunneling,
3. two-path interference.

In each case, Callender argues that TQM does not provide us with the kind of core description common to all versions of QM that would be required for a non-speculative realism.

The first part of my reply to these objections is to concede that some postulates that are widely recognized as elements of TQM may not be apt for realist interpretation. Just like any other contemporary scientific realism, realism about TQM needs to be selective in its commitments. One obvious motivation to withhold such commitment obtains if the textbooks themselves caution us against taking a certain description at face value. This seems to be the case for well-known examples of semi-classical orbits; I know of no serious QM textbook that speaks of electrons “orbiting” the nucleus of an atom without adding some qualification that such talk is not to be taken literally. It is then neither surprising nor problematic for the realist that these orbits do not agree with the (speculative but literally intended) description of how Bohmian particles (let alone GRW flashes or matter density) behave.

A similar remark can be made with respect to Callender’s third example (the two-path interference experiment), although in that case, the textbooks are generally less cautious about treating talk of particle trajectories literally. Indeed, Callender (2020, 70) is right to point out that the whole debate on allegedly “surrealistic trajectories” in Bohmian mechanics could only get started because the authors of the original attack (Englert et al. 1992) assumed that TQM tells us something about the “real” trajectories.^18^ Some analysis is therefore needed to arrive at the proper assessment of what we should be realists about, given the TQM account of the two-path experiment. And it is precisely this kind of analysis that has been undertaken by defenders of Bohmian mechanics, in order to show that the trajectories predicted by their theory are not actually in contradiction with TQM (because the latter does not really say anything about particle trajectories), but only with some pretheoretical assumptions about what is measured in a position measurement.

Before turning to the second example on Callender’s list, let me take a step back and admit that my response so far has not yet identified any positive ontological content of TQM; it has merely shown that a properly selective realism about TQM need not entail any problematic ontological commitments. But is there any positive ontology to be found in the textbook treatment of these cases? I suspect that the answer is “no” for the case of semi-classical orbits, but I will now argue for an affirmative answer in the other two cases.

Discussing the phenomenon of quantum tunneling, Callender stresses the discrepancy between “the minimal implication found in the textbooks on the most canonical system, namely, that *something is reflected*” and the Bohmian treatment, according to which “there is nothing *reflected* at all” (69). Again, the proper response to this problem starts by being appropriately selective in one’s realism about TQM, but this time, this first (negative) part of the response immediately leads to a positive statement of TQM realism. As it turns out, Callender himself (in a footnote) indicates how this is to be reached:

I admit that this example is an artifact of an inadequacy of the textbook treatment of tunneling. The normal textbook treatment via plane waves is flawed: it’s not clear how such stationary states justify talk of entities moving from the left and so on; worse, these waves are not renormalizable, so they aren’t physical. A better treatment is possible (see Norsen 2013). (69n2)

This “better treatment” replaces the (unphysical) plane waves with normalizable wave packets and is therefore clearly the account to be favoured when it comes to drawing ontological conclusions from TQM. Now one of the upshots of Norsen’s account is that the discrepancy noted by Callender disappears, because within this approach, some Bohmian particles (along with a part of the wave packet) are indeed reflected at the potential barrier. This is, however, not the most important point for our present purposes, because of course, the Bohmian particles are still not part of the non-speculative ontology we are after. Instead, the crucial aspect of Norsen’s paper is the indispensability of the wave packets even for a Bohmian treatment of this phenomenon, which suggests that it is these wave packets that are the proper target of a non-speculative ontological commitment, in the same way as spin was argued to be such a target in the previous section.^19^ Let me stress again that the success of this response does not depend on the fact that the motion of the Bohmian particles in this case happens to coincide with the motion of the wave packets, but on the fact that (as explained in Section 4.2) any Bohmian who seeks to resist a rather general kind of instrumentalism should endorse some kind of realism about the wave function. This becomes important when we now turn back to Callender’s third example.

### The *nothing-there* objection

Just as in the case of tunneling, wave packets play an essential role not only in the TQM approach to the two-path experiment, but in the Bohmian approach as well (cf. Barrett 2000). On the same grounds as before, I therefore consider these wave packets as part of quantum ontology. This, however, raises a worry that may have lingered in the mind of the reader since Section 3, but becomes particularly pressing in the context of this example. I call it the *nothing-there* objection, and it stems from the potentially dubious ontological status of the wave function in Bohmian mechanics (and other PO theories). Even if my argument in Section 4.2 has succeeded in convincing the Bohmian that some reality must be granted to the wave function, she might still insist that the wave function is not and cannot give rise to something that *is there* (or, in the jargon of the debate, to a “local beable”).^20^ One problem is that the wave function in general lives in a high-dimensional configuration space instead of ordinary space, but even in the one-particle case, one might deny local beable status to the wave function, such that there still seems to be an underdetermination: according to my TQM-based story, there is a wave packet on each of the two paths, whereas according to the Bohmian story, there is a particle on one path, and simply nothing on the other.

In my view, this objection rests on an implausible separation of ontology from dynamics that one often encounters in presentations of the PO approach and which has been forcefully criticized in a recent paper by Wayne Myrvold:

The most serious danger [of the PO approach], though, it seems to me, is the danger of sliding into thinking that there is a two-step process. *First,* one posits an ontology, with no dynamical assumptions, that is, no assumptions about how it behaves, and *then* one posits a dynamics for it. … The problem with this is that, until we have said something about how the purported ontology acts, we haven’t yet given sense to the claim that it is there at all. What it is for an object to occupy a region of space, or, indeed, to have any sort of spatial relations to anything, is for it to do something there—exclude other objects, or reflect light, or something of the sort. (Myrvold 2018, 104–105)

The inadequacy of separating ontology from dynamics becomes particularly salient in the delayed-choice variant of the two-path experiment, in which a detector is used to determine which path the particle has taken (see Barrett 2000, sec. 5 for details). Due to the non-local dynamics of Bohmian mechanics, it can happen that a particle triggers a detector located on the path it has *not* taken! To use Myrvold’s terminology, the particle does not really occupy the region of space in which the theory says it is, because the region where it does something (in this case, trigger a detector), is somewhere else. Granted, the theory has a coherent story to tell about the particle’s location and the non-local effect it has on the detector, but it is only in a highly technical sense that the particle “is there” while there is allegedly “nothing there” where it produces its effect. If we want to hold on to the ordinary sense of “being there” from which the *nothing-there* objection derives its force, we should acknowledge that there is something where the detector is triggered. This is the so-called empty wave packet, which is just what TQM postulates as well.

One might think that this response fails in the multi-particle case, because the wave packets are then not located in ordinary space, but in configuration space. However, this would again be to insist on an inappropriate notion of “location”. Insofar as the wave packets affect detectors (or other ordinary objects), they do indeed occupy regions of ordinary space, hence they “are there” in the ordinary sense of the term. Incidentally, the wave packet’s *being there* in virtue of its *doing something there* is yet another illustration of the kind of functional ontology introduced in Section 3.1.

This functionalism-inspired identification of *being there* with *doing something there* will strike some readers as too strong, as it essentially makes action at a distance metaphysically impossible.^21^ I agree that action at a distance should not be ruled out *a priori* (not least because the realism about wave-function collapse defended in Section 4.3 in some situations involves a kind of action at a distance), so perhaps the above identification should be replaced by a one-way conditional, such that *doing something there* is only a necessary, but no longer a sufficient condition for *being there*. As far as I see, this does not undermine realism about the empty wave packet. Consider first the multi-particle case just discussed. Here the cost of denying the existence of a wave packet at the location of the detector goes well beyond the simple acknowledgment of action at a distance, because it is not even clear what should be meant by the “distance” between the wave packet in configuration space and the detector in ordinary space. Let us therefore focus our attention on cases of action between objects in ordinary space. Traditionally, the most prominent motivation for countenancing the possibility of action at a distance is Newtonian gravity (to the extent that one considers a Newtonian world metaphysically possible). I am sympathetic to that motivation, but I do not think it translates very well to the case of the Bohmian account of the two-path experiment, so as to obviate the need for there being an empty wave packet. The first difference between the two cases is that the field theoretic perspective is optional in Newtonian gravity (see Section 3.2), whereas it is mandatory in Bohmian mechanics in the sense that one cannot do without the wave function (see Sections 4.2 and 5.1). This does not yet force us to reify the empty wave packet in the two-path scenario, because one can insist that the real action on the detector is exerted (at a distance) by the Bohmian particle. But then notice the second difference between Newtonian gravity and Bohmian mechanics: While both are cases of particles acting where they are not, the action in the Newtonian case directly depends (according to the law of gravity) on the acting particle’s position, whereas the particle’s being *there* (as opposed to somewhere else) does not matter in the Bohmian case, except by determining which wave packet is relevant for the reaction of the detector. This reaction therefore depends just as much on the wave packet’s *being there* as on the particle’s.

To sum up, the separation of ontology from dynamics on which the *nothing-there* objection rests does not do justice to the explanatory role of the wave function in the two-path experiment. Insofar as this means that any reasonable account of QM needs to admit at least some aspects of the wave function into its ontology, there is no serious underdetermination (“serious” in the sense of Section 1) with respect to them, and consequently, they form part of the non-speculative ontology of QM. On the other hand, if one insists that a quantum ontology devoid of the wave function is reasonable enough to generate a serious kind of underdetermination, then this part of my case for a non-speculative quantum ontology admittedly remains incomplete at the level of non-relativistic QM. The assessment of what happens in QFT is beyond the scope of this paper, but there are grounds for optimism: One does not need to go as far as Wallace (cited in Section 2) in discounting the prospects of PO approaches to QFT, it suffices to realize that even within these approaches, the explanatory work is predominantly performed by the dynamical variables, which makes the restriction of ontological status to nothing but the PO even more questionable than in non-relativistic QM. Accordingly, the *nothing-there* objection becomes progressively questionable in QFT as well.

### The vagueness objection

A last line of criticism (which is essentially a generalization of Saatsi’s critique introduced in Section 4.1) is that my approach is purchasing what looks like ontological commitment at the price of vagueness. Earlier in the present section and in the previous one, I repeatedly considered different approaches to QM which (in contrast to TQM) suggest candidates for a fundamental ontology: Bohmian, Everettian and collapse theories. (I will subsume them under the label “fundamental candidates” for what follows.) My claim was that the adherents of all these approaches can agree on certain commitments concerning the reality of spin, wave function collapse and wave packets. Now this may turn out to be a purely verbal agreement without any ontological significance, because words like “spin” or “collapse” mean radically different things according to the different fundamental candidates. The vagueness (or ambiguity) of such terms, so the objection goes, creates the illusion of a common ontology, while there really isn’t any ontological common ground on which to build a non-speculative quantum ontology.

The allure of this objection depends on what I take to be a false view of the relationship between TQM and the various fundamental candidates. The supposed ambiguity of terms like “spin”, “collapse” and “wave packet” only obtains under the assumption that what they *really* mean is not given by TQM, but by one of the fundamental candidates (while underdetermination prevents us from knowing which one). And this assumption in turn stems from the view that TQM should ultimately be *replaced* by one of the fundamental candidates. However, the examples considered in this paper do not support the idea of TQM being replaced by one of the fundamental candidates. For the case of spin, we saw in Section 4.2 that even a view like Super-Humeanism, which seeks to exclude spin from its ontology, cannot afford to deny what TQM says about spin. The same holds with respect to the TQM account of wave packets in the cases of quantum tunneling (Section 5.1) and two-path interference (Section 5.2). Finally, Section 4.3 showed that a notion of wave function collapse familiar from TQM survives even if one takes a no-collapse theory like Everettian QM to govern the fundamental level of reality.

Of course, this kind of persistence of TQM can always be viewed as a purely practical matter without any ontological import. This brings us back to the meta-ontological considerations mentioned in Section 3. I do not have a knock-down argument against the fundamentalist approach to ontology (since non-question-begging arguments for or against philosophical stances in general are hard to come by, see Chakravartty (2017, chs. 7 and 8)), but I hope that the cases discussed here suffice to highlight its costs: The more one denies ontological import to non-fundamental posits like spin and wave packets, the more one dissociates ontology from what actually performs the explanatory and predictive work of science. In short, one withdraws from the very idea of scientific ontology.

Such withdrawal has an equally unflattering counterpart in the area of semantics, from which the vagueness objection takes its starting point. As mentioned above, the fundamentalist takes the real meaning of terms employed within TQM to be given by the fundamental theory which is ultimately supposed to replace it. By contrast, what I take to be the appropriate approach to semantics is a functionalist one, in line with the functionalist approach to ontology described in Section 3. The meaning of “spin” etc. is thus determined by the way in which the notions are employed in TQM and its applications, and this will not change even if one takes TQM to be theoretically reducible to one of the fundamental candidates, because such a reduction does not entail the disappearance of TQM. Note, by the way, that this view of semantics is by no means a recent invention, but is already present in Nagel’s (1961, 357) classic account of theory reduction, when he criticizes “the unwitting double talk” of those who suppose that the reduction of thermodynamics to the kinetic theory of heat involves a redefinition of the term “temperature”, such that it *now* means “mean kinetic energy”. To adopt the functionalist view also saves us from the awkward conclusion that those practicing scientists who successfully employ the notions of TQM on a daily basis (without giving much thought to the fundamental candidates) do not *really* know what these notions mean.

But is TQM by itself really sufficient to endow the notions in question with a precise meaning? Let us have a last look at my three examples with regard to this question. The mathematical description of spin in TQM can hardly be accused of imprecision, but the fundamentalist will complain that such mathematical precision only implies ontological precision if a clear interpretation of the mathematical apparatus is given. The functionalist agrees, but rejects the fundamentalist’s requirements on a “clear interpretation”: as long as TQM precisely informs us about how quantum systems behave as a function of their spin state (which it certainly does, see Section 4.1), it yields all the ontological precision one can expect from an effective theory like QM.

In contrast to the case of spin, there is no precise mathematical definition of “wave packet”. The boundary between what counts as a wave packet and what is some other structure in the wave function is vague, and since I have only given arguments for realism about wave packets, not about the wave function in general, I must admit that the corresponding ontological commitment has a vague boundary as well. This is not a problem, however. Sections 5.1 and 5.2 described clear cases of wave packets and argued for (non-speculative) realism about them. To what extent realism about boundary cases or other structures in the wave function is justified can be left open for future inquiry.

The notion of wave function collapse indeed suffers from an insuperable vagueness in TQM, due to its dependence on the anthropocentric notion of measurement. TQM by itself does not have the resources to overcome this dependence, so this is indeed a case in which the precise meaning of a term needs to be determined by one of the fundamental candidates. As we saw in Section 4.3, these candidates make incompatible claims about the occurrence of collapse on the fundamental level, but we also observed a striking convergence of how they describe collapse on the effective level. Thanks to this convergence, the functionalist has a sufficiently precise notion of wave function collapse at his disposal, which allows him to overcome the vagueness of the corresponding TQM notion without settling for any particular one of the (speculative) fundamental candidates.

This concludes (for the time being) my account of a non-speculative ontology for QM. The hitherto unsuccessful quest described in Section 2 has therefore finally met with some success, but it is, of course, far from complete. One possible direction for further progress is opened by the inherently non-fundamental character of this ontology, as described in Section 3. It is not excluded that future research might reduce the underdetermination between the different variants of QM and thereby reduce the degree of speculation involved in committing oneself to any one of them. It is, however, unlikely that the underdetermination will completely disappear, hence I suspect that the kind of non-fundamental (and, as mentioned at the end of Section 4, necessarily incomplete) ontology proposed here will remain the best we can do. It thus seems worthwhile to further develop this approach, taking account of further aspects of TQM beyond the few examples I was able to discuss in this article. There are still ontological lessons to be learnt from QM, even if we refuse to engage in speculation.

## Footnotes

1 Within other approaches, this kind of ontological diversity is somewhat less obvious, but it is present nonetheless. For example, although Bohmian mechanics is usually associated with a PO of particles, a non-PO version of it has been defended (Albert^2015^, chs. 6 and 7). Conversely, there are reasons to formulate the many-worlds theory by including a PO (Allori et al. 2011), although most Everettians prefer not to do so.

2 Stanford then goes on to argue for a kind of underdetermination that *should* be taken seriously in philosophy of science, based on the possibility of *unconceived alternatives* to current scientific theories. I can set this problem aside for the reason just mentioned: it is a problem that concerns fundamental theories in all scientific domains, while I am here only interested in underdetermination insofar as it specifically affects quantum theories.

3 Callender (2020, sec. 4.6) contains a more elaborate (and beautifully framed) description of the situation, discussing the same two authors.

4 I learnt this surprising fact from Wallace (2020, 79n2), to whom I also owe the terminological suggestion of distinguishing “representationalist” approaches from “non-representationalist” ones.

5 I am indebted to an anonymous referee for helping me clarify my understanding of (non-)representationalism.

6 An anonymous referee has raised the question whether I think that it should be informed *exclusively* by our best current theories. The answer is a clear *no*. I am very sympathetic to the tradition of *experimental realism*, which emphasizes the direct ontological import of experimental work, independently of any inference from theory to reality (Egg 2018). I realize, however, that this is a minority position, especially among philosophers of quantum physics. Therefore, I seek to keep my quantum ontology as independent as possible from any commitment to experimental realism. This includes recognizing the role of theory even for notions which I take to connect to reality predominantly through experiment, as is the case for the examples discussed in this paper. However, my concession to theory realism can only go so far; admittedly, I need to adopt a rather loose notion of “theory” in order for it to apply to TQM.

7 There are various notions of fundamentality in the contemporary metaphysical debate (see Tahko 2018 for an overview), but the only distinction that matters for the present context is the one between relative and absolute fundamentality. On any absolute understanding of fundamentality, it is wrong to expect fundamental ontology to be informed by a non-fundamental theory like QM.

8 There is an important difference, of course: we do not (yet) know from what kind of theory our present QFTs emerge, whereas we do know quite well how QM emerges from QFT (see, e.g., Myrvold 2015). The striking feature of the effective field theory programme is that the lack of knowledge about the underlying theory does not impair the reliability of the effective theory. In what follows, I will treat QM as an effective theory as well, in the sense that how it emerges from QFT won’t matter for my discussion of its ontology.

9 See Wallace (2020, sec. 5.6) for further criticism of “discussing nonrelativistic quantum particle mechanics *under the fiction that it is a fundamental and universal theory*”.

10 This should not be taken as a response to a Laudan-style historical argument against scientific realism (in which context this example is most often discussed). The fact that phlogiston theory is not an effective theory in the relevant sense may not remove the force (if such there is) of a historical argument, because that fact may (at least for a certain period) not have been epistemically accessible to researchers at the time.

11 This is also the reason why one cannot simply bypass Ruetsche’s objection by restricting effective realism to *currently accepted* theories (as opposed to allegedly *superseded* theories like Newtonian gravitation). Bokulich’s example demonstrates that Newtonian gravitation still is our *best current* theory for some relevant domains, and it is therefore a proper target for effective realism.

12 Letter from Newton to Richard Bentley, quoted from Janiak (2007, 128). Interestingly, Janiak shows how Newton, despite not yet having the field concept at his disposal, could still maintain realism about gravitational forces while rejecting action at a distance.

13 There is a relevant difference between the (Newtonian) gravitational and the electromagnetic field, in that the latter propagates with a finite speed while gravity is supposed to act instantaneously. But even this type of action at a distance can be removed from Newton’s theory without compromising the reality of gravitational forces (see, e.g., the semi-Newtonian models of gravity discussed in Walter (2007)).

14 For reasons of exposition, I focus on the spin part for the moment. I also endorse a (properly selective) realism about the spatial part of the wave function, which will be discussed in Section 5.

15 There are versions of the Bohmian approach that stay closer to the TQM picture by viewing spin as an intrinsic property of the particles (e.g., Bohm and Hiley 1993, ch. 10). In keeping with my methodology sketched in the previous paragraph, I will disregard this variant, which also does not seem to have many supporters among contemporary Bohmians.

16 This simple presentation glosses over a few subtleties. First, the exact point at which the number of involved particles gets large enough to (practically) ensure a GRW collapse depends on the specific experimental setup. In some cases, the collapse may not occur before a visual signal gets recorded in the nervous system of an observer (Aicardi et al. 1991). Second, one might worry that the different ontologies that can be associated with the GRW theory (matter density, flashes, no PO) create an additional underdetermination. It seems, however, that this can be subsumed under the above discussion of spin in Bohmian mechanics, given the structural similarities between Bohmian mechanics and the PO-versions of GRW (Allori et al. 2008). Finally, ongoing discussion of the so-called “tails problem(s)” (McQueen 2015) may sustain the worry that collapse theories do not actually succeed in discarding superpositions. To the extent that this worry is cogent, it diminishes the ontological difference between GRW and Everett, and therefore attenuates the underdetermination problem to be discussed here.

17 See Rosaler (2016) for a technical account of how decoherence ensures an effective collapse of the wave function, which is neutral with respect to the differing views on collapse (or absence thereof) at the fundamental level.

18 See Solé (2017) for an up-to-date account of that debate. The lessons that are relevant for present purposes can, however, already be found in Barrett (2000).

19 I wish to remain neutral on wider applications of such indispensability considerations, in particular with respect to the indispensability of mathematics. Therefore, my claim is only that indispensability responds to the kind of underdetermination worry that concerns us here, not that it is, in and of itself, a sufficient condition for realism.

20 See Egg (2017) for a general defense of the idea that some local beables can be derived from the wave function.

21 I am indebted to an anonymous referee for drawing my attention to this consequence.
